# Propofol Attenuates Small Intestinal Ischemia Reperfusion Injury through Inhibiting NADPH Oxidase Mediated Mast Cell Activation

**DOI:** 10.1155/2015/167014

**Published:** 2015-07-12

**Authors:** Xiaoliang Gan, Dandan Xing, Guangjie Su, Shun Li, Chenfang Luo, Michael G. Irwin, Zhengyuan Xia, Haobo Li, Ziqing Hei

**Affiliations:** ^1^Department of Anesthesiology, The Third Affiliated Hospital, Sun Yat-sen University, Guangzhou 510630, China; ^2^Zhongshan Ophthalmic Center, Department of Anesthesiology, Sun Yat-sen University, Guangzhou 510060, China; ^3^Department of Anesthesiology, University of Hong Kong, Hong Kong

## Abstract

Both oxidative stress and mast cell (MC) degranulation participate in the process of small intestinal ischemia reperfusion (IIR) injury, and oxidative stress induces MC degranulation. Propofol, an anesthetic with antioxidant property, can attenuate IIR injury. We postulated that propofol can protect against IIR injury by inhibiting oxidative stress subsequent from NADPH oxidase mediated MC activation. Cultured RBL-2H3 cells were pretreated with antioxidant N-acetylcysteine (NAC) or propofol and subjected to hydrogen peroxide (H_2_O_2_) stimulation without or with MC degranulator compound 48/80 (CP). H_2_O_2_ significantly increased cells degranulation, which was abolished by NAC or propofol. MC degranulation by CP further aggravated H_2_O_2_ induced cell degranulation of small intestinal epithelial cell, IEC-6 cells, stimulated by tryptase. Rats subjected to IIR showed significant increases in cellular injury and elevations of NADPH oxidase subunits p47^phox^ and gp91^phox^ protein expression, increases of the specific lipid peroxidation product 15-F_2t_-Isoprostane and interleukin-6, and reductions in superoxide dismutase activity with concomitant enhancements in tryptase and *β*-hexosaminidase. MC degranulation by CP further aggravated IIR injury. And all these changes were attenuated by NAC or propofol pretreatment, which also abrogated CP-mediated exacerbation of IIR injury. It is concluded that pretreatment of propofol confers protection against IIR injury by suppressing NADPH oxidase mediated MC activation.

## 1. Introduction

Small intestinal ischemia reperfusion (IIR) injury has been emerged in many pathophysiological settings, including septic and hemorrhagic shock induced hypoperfusion [1], as well as acute mesenteric ischemia [2] and small intestine transplantation or liver resection [3]. IIR remains to be a critical problem associated with high mortality [4].

During IIR, oxidative stress is increased due to burst production of reactive oxygen species (ROS), which is a major mechanism of IIR injury [5]. Numerous studies have revealed that increased ROS production resulted from overactivation of the prooxidant enzyme NADPH oxidase, which abundantly exists in intestine tissue [6] and plays critical roles in mediating tissue injury related to a range of inflammatory diseases [7], including ischemia reperfusion injury [8, 9]. Inhibition of NADPH oxidase by N-acetylcysteine (NAC), a scavenger of oxygen radicals, has been shown to greatly attenuate myocardial ischemia reperfusion injury [10, 11]. However, the mechanism governing ROS production, specifically, from overactivation of NADPH oxidase, during IIR is yet to be explored.

Activation of NADPH oxidase can lead to mast cells (MC, cells that widely present throughout small intestine) degranulation/activation [12], wherever MCs activation has been demonstrated to be a key mediator in the pathogenesis of IIR and contributing to many disorders as a result of increased release a diverse range of mediators including histamine, tryptase, and inflammatory cytokines such as interleukin-6 (IL-6) [13] and that inhibition of MCs from degranulation can alleviate IIR injury [14, 15]. We have previously found that ROS production was significantly increased after IIR which is parallel with the enhancements in MC degranulation [16], but it is unknown whether a causal relationship exists between increased ROS production and MC degranulation in the setting of IIR* in vivo*. These findings [6, 17] together with reports showing that MC can be activated in a ROS-dependent pathway [18], both* in vitro* [18] and* in vivo* [16], prompted us to postulate that during IIR increased ROS production initiates and/or exacerbates IIR injury primarily via activating MC and that NADPH oxidase activation is increased during IIR which may be a major source of ROS overproduction during IIR.

Propofol, an intravenous anesthetic with antioxidant property that we widely used in intensive care unit and operation theatre, has been shown to dose-dependently attenuate myocardial ischemia reperfusion injury in patients [19]. Propofol has also been shown to inhibit mast cell exocytosis in a dose-dependent manner* in vitro* [20]. A most recent study shows that propofol attenuates brain trauma induced cerebral injury through inhibiting NADPH oxidase activation [21]. We, therefore, hypothesized that inhibition of ROS mediated MC activation subsequent to attenuation of intestinal NADPH oxidase activation may represent a major mechanism by which propofol attenuates IIR injury. This hypothesis was tested in a rat model of mesenteric ischemia reperfusion* in vivo* and a rat cell line of mast cell exposed to ROS* in vitro*.

## 2. Materials and Methods

### 2.1. Cell Culture

Rat mast cell line (RBL-2H3) was purchased from the cell bank of the Chinese Academy of Sciences (Shanghai, China) and was cultured in Eagle's minimum essential medium containing 10% fetal bovine serum, supplemented with 100 U/mL penicillin and 100 *μ*g/mL streptomycin at 37°C in a humidified atmosphere of 5% CO_2_ as described [22]. A rat small intestinal epithelial cell line (IEC-6) was also obtained from the cell bank of the Chinese Academy of Sciences (Shanghai, China). This cell line was cultured in 1640 RPMI medium with 10% fetal bovine serum at 37°C in a 5% CO_2_ incubator.

### 2.2. Measurement of Cell Degranulation

Degranulation of RBL-2H3 cells was measured by determining the activity of released *β*-hexosaminidase in the culture supernatants and cell lysates [23]. The RBL-2H3 cells were incubated with different concentrations of NAC [24] or propofol [20] for 12 h; after the cells were washed with 1 × PBS for 3 times, RBL-2H3 cells were incubated in a 24-well plate (1 × 10^6^ cells/well) at 37°C overnight. The above cells were washed with 1 × PBS and then incubated with different concentrations of lipopolysaccharides (LPS) [25] or H_2_O_2_ [26] or Compound 48/80 [27] for 1 h or 4 h. To measure the amount of *β*-hexosaminidase activity released from the cells, the cultured media were transferred and centrifuged at 4°C. The supernatant (25 *μ*L) was mixed with 50 *μ*L p-NAG (10 mM) in 0.1 M sodium citrate buffer (pH 4.5) into a 96-well plate and incubated for 1 h at 37°C. The reaction was terminated by the addition of stop buffer (0.1 M sodium carbonate buffer, pH 10.0). The *β*-hexosaminidase activity was determined by measuring the difference of absorbance at wavelength 405 nm.

### 2.3. Measurement of Cell Viability

IEC-6 cells were seeded into 96-well flat bottom culture plates at a density of 5 × 10^4^ cells/well and incubated for 24 h. After exposure to tryptase (0–1000 ng/mL) for 12 h in RPMI 1640 culture medium, the cell viability was measured by modified MTT assay as described [28]. The assay detected the reduction of the tetrazolium to formazan product. The cell viability was evaluated using the following formula. Survival rate (%) = (*A*_sample_ – *A*_blank_)/(*A*_control_ – *A*_blank_) × 100%.

### 2.4. Assessment of Cell Cytotoxicity

Cytotoxicity (cell necrosis) was assessed by measuring the release of lactate dehydrogenase (LDH). After exposure of the cells to various concentrations of tryptase (0–1000 ng/mL) for 2 h in RPMI 1640 culture medium, cell necrosis was assessed by LDH-release assay using a commercial kit (Jiancheng, China) according to the manufacturer's instruction.

### 2.5. Animals

Female Sprague-Dawley (SD) rats weighing 180–200 g, purchased from Animal Center of Guangdong Province (Guangzhou, China), were housed individually in wire-bottomed cages and were placed under pathogen free condition illuminated from 8:00 AM to 8:00 PM (12:12 h light-dark cycle) for one week before use. The experimental protocol and design was approved by the Sun Yat-sen University Animal Experimentation Committee and performed according to Sun Yat-sen University Guidelines for Animal Experimentation.

### 2.6. *In Vivo* Rat IIR Model and Treatments

All the animals were fasted for 16 h (while free access to water was allowed) before surgery. Rats were, respectively, injected with N-acetylcysteine (NAC, 0.5 g/kg, from Sigma company), propofol (50 mg/kg, commercial product Diprivan from AstraZeneca), intralipid (50 mg/kg, 20%, emulsion from Sigma), or normal saline (0.5 mL/100 g), which served as the control group, intraperitoneally at 6:00 PM for 3 successive days. The dosages of NAC were chosen based on the results showing that treatment of rats with i.p. NAC (500 mg kg^−1^ per day for 9 days) improved the renal hemodynamic changes triggered by cisplatin-mediated nephrotoxicity [29]. The dose of propofol was chosen based on the finding that propofol 50 mg/kg given intraperitoneally provided sedative effect but not anesthetic effect [30] and that propofol when used at this dosage attenuated IIR injury in rats [31]. At the 4th day, parts of the rats were sacrificed by overdose of anesthetic chloral hydrate, and the intestinal mucous was obtained and scraped for further determination and the intestinal morphological changes were assessed.

### 2.7. Experimental Groups

The other rats were divided into the following groups.

1. Sham-operated group (SHAM) (*n* = 6): rats pretreated with normal saline (10 mL/kg, i.p.) were subjected to identical surgical procedures except for superior mesenteric artery (SMA) occlusion for 75 min and were kept under anesthesia during the experiment and were administrated with the same volume of normal saline (1 mL/kg, i.v.) as reagent solvent control.
2. Sole IIR group (IIR) (*n* = 6): rats pretreated with normal saline (10 mL/kg, i.p.) were subjected to small intestinal ischemia by occluding SMA (75 min), followed by reperfusion (2 h) plus administration of normal saline (1 mL/kg, i.v.) 5 min immediately before reperfusion.
3. IIR + Compound 48/80 group (IIR + CP) (*n* = 12): rats pretreated with normal saline (10 mL/kg, i.p.) were subjected to small intestinal ischemia by occluding SMA (75 min), followed by reperfusion (2 h) plus administration of Compound 48/80 (0.75 mg/kg, i.v.) dissolved in normal saline (1 mL/kg) 5 min immediately before reperfusion.
4. NAC + IIR group (NAC + IIR) (*n* = 6): rats pretreated with NAC (0.5 g/kg, i.p./day) dissolved in normal saline (10 mL/kg) for 3 successive days were subjected to small intestinal ischemia by occluding SMA (75 min), followed by reperfusion (2 h) plus administration of normal saline (1 mL/kg, i.v.).
5. NAC + IIR + Compound 48/80 group (NAC + IIR + CP) (*n* = 6): rats pretreated with NAC (0.5 g/kg, i.p.) were subjected to small intestinal ischemia by occluding SMA (75 min), followed by reperfusion (2 h) plus administration of Compound 48/80 (0.75 mg/kg, i.v.).
6. Propofol + IIR group (Pro + IIR) (*n* = 6): rats pretreated with propofol (50 mg/kg, i.p./day) dissolved in intralipid (10 mL/kg) for 3 successive days were subjected to small intestinal ischemia by occluding SMA (75 min), followed by reperfusion (2 h) plus administration of normal saline (1 mL/kg, i.v.).
7. Propofol + IIR + Compound 48/80 group (Pro + IIR + CP) (*n* = 6): rats pretreated with propofol (50 mg/kg, i.p.) were subjected to small intestinal ischemia by occluding SMA (75 min), followed by reperfusion (2 h) plus administration of Compound 48/80 (0.75 mg/kg, i.v.).
8. Intralipid + IIR group (Lip + IIR) (*n* = 12): rats pretreated with intralipid (50 mg/kg, i.p.) (with a volume of 10 mL/kg) were subjected to small intestinal ischemia by occluding SMA (75 min), followed by reperfusion (2 h) plus administration of normal saline (1 mL/kg, i.v.).
9. Intralipid + IIR + Compound 48/80 group (Lip + IIR + CP) (*n* = 12). rats pretreated with intralipid (50 mg/kg, i.p.) were subjected to small intestinal ischemia by occluding SMA (75 min), followed by reperfusion (2 h) plus administration of Compound 48/80 (0.75 mg/kg, i.v.).

It is of notice that, with respect to the low survival rate in rats that underwent IIR in the presence of CP, experiments were performed on a total of 12 animals in IIR + CP and IIR + CP + intralipid groups. In all the experimental groups, the rats were anesthetized by intraperitoneal injection of 10% chloral hydrate (3.5 mL/kg) after fasting for 16 h. The Compound 48/80 (Sigma, USA; 0.75 mg/kg) or the same volume of physiological saline was intravenously injected via the tail vein at 5 min before reperfusion. The doses of agents were adjusted in accordance with our previous study [15]. During the surgery, all the rat body temperature was maintained at 38°C using heated pad. And 10 mL/kg 37°C normal saline was injected subcutaneously to avoid dehydration after the abdomen had been closed.

### 2.8. Collection of Intestinal Mucosa

Upon the completion of the abovementioned treatments and experiments, the rats were anesthetized and then euthanized. The whole small intestine was removed carefully, and a segment of 1.0 cm intestine (located at 10 cm away from the terminal ileum) was cut and fixed in 10% formaldehyde and then embedded in paraffin for section. The remaining small intestine was washed thoroughly with 0°C normal saline and then opened longitudinally to expose the intestinal epithelium, after being rinsed completely with 0°C normal saline and dried with suction paper. The mucosal layer was harvested by gentle scraping of the epithelium with a glass slide with a plate on the ice and then was stored at −70°C for further measurements.

### 2.9. Intestinal Histology

Five *μ*m thick sections were prepared from paraffin-embedded intestine tissue; the segment of small intestine was stained with hematoxylin-eosin. And the damage of intestinal mucosa was evaluated by two histologists blinded to the experiment according to Chiu's standard [32]: Grade 0, normal mucosa; Grade 1, development of subepithelial Gruenhagen's space at the tip of villus; Grade 2, extension of the space with moderate epithelial lifting; Grade 3, massive epithelial lifting with a few denuded villi; Grade 4, denuded villi with exposed capillaries; Grade 5, disintegration of the lamina propria, ulceration, and hemorrhage.

### 2.10. Detection of 15-F_2t_-Isoprostane Content in the Small Intestinal Mucosa

Small intestinal mucosa was homogenized with normal saline. The tissue content of free 15-F_2t_-isoprostane, an index of* in vivo* oxidative stress-induced lipid peroxidation, was measured using commercial immunoassay kits (Cayman Chemical, Ann Arbor, MI) as we described [33].

### 2.11. Assay of Superoxide Dismutase (SOD) Activity and Hydrogen Peroxide Content in Small Intestinal Mucosa

Small intestinal mucosa was made into a homogenate with normal saline, frozen at −20°C for 5 min, and centrifuged for 15 min at 4000 r/min. Supernatants were transferred into fresh tubes for evaluation of SOD activity and hydrogen peroxide content. SOD activity and hydrogen peroxide content were assessed by SOD and hydrogen peroxide detection kits according to the manufacturer's instructions (Jiancheng Bioengineering Ltd, Nanjing, China). Presented data were normalized to tissue weight.

### 2.12. Measurement of *β*-Hexosaminidase Level in Serum

*β*-hexosaminidase level in the serum was determined using modification of a previously described method [34]. Briefly, 50 *μ*L of serum was incubated with 50 *μ*L of 1 mM p-nitrophenyl-N-acetyl-*β*-D-glucosaminide dissolved in 0.1 M citrate buffer (pH 5) in a 96-well plate at 37°C for 1 h. The reaction was terminated with 200 *μ*L/well of 0.1 M carbonate buffer (pH 10.5). The absorbance at 405 nm was measured using a Microplate reader.

### 2.13. Western Blotting

Intestinal mucosa samples were homogenized with lysis buffer for 30 seconds in a mortar and pestle with liquid nitrogen. Homogenates were centrifuged at 13000 rpm for 10 min at 4°C and the supernatant was collected as the source of protein sample. The proteins were processed with standard methods for Western blot analysis as described [10]. Rat monoclonal anti-tryptase antibody, rat monoclonal anti-gp91^phox^ and anti-p47^phox^ antibodies, rat monoclonal anit-P-selectin and anti-ICAM-1 antibodies, and *α*-tubulin antibody were obtained from Santa Cruz (Santa Cruz, CA, USA). The secondary antibody conjugated to horseradish peroxidase was diluted at 1 : 2,000 (Santa Cruz, USA). Immunoblots were incubated with an enhanced chemiluminescence detection system (KeyGen Biotech, China) and the densitometry analysis was performed using Quantity One software.

### 2.14. Determination of IL-6 Production in Small Intestinal Mucosa by Enzyme Immunoassay

Intestinal mucosa tissues were made into homogenates with frozen normal saline and spun at 4000 r/min for 15 minutes. Supernatants were then transferred into fresh tubes for detection of IL-6 contents. Briefly, intestinal protein was measured by BCA Protein Assay Kit provided by KenGen Biotech Company, Nanjing, China, and the protein contents were expressed as g/L. The levels of IL-6 were measured using commercial ELISA kits following manufacturer's instructions (R&D systems Inc, USA). The absorbance was read at 450 nm by a Biokinetics microplate reader Model EL340 (Biotek Instruments, USA), and the results were expressed as ng/L; then the final levels of IL-6 in the intestine were calculated as ng/g protein.

### 2.15. Determination of Myeloperoxidase (MPO) Activity in Small Intestinal Mucosa

Myeloperoxidase (MPO) activity was determined with the O-dianisidine method [35], using a MPO detection kit (Nanjing Jiancheng Bioengineering Institute) as we described [36]. MPO activity was defined as the quantity of enzyme degrading 1 *μ*mol of peroxide per minute at 37°C and was expressed in units per gram weight of wet tissue.

### 2.16. Statistical Analysis

The data (except for the survival rates) were expressed as Mean ± SEM. All biochemical assays were performed in duplicate. Analysis of variance was performed using Graphpad Prism software. One-way analysis of variance was used for multiple comparisons, followed by Bonferroni's Student's *t*-test for unpaired values. The survival rate was expressed as the percentage of live animals, and the Mantel Cox log rank test was used to determine differences between groups. Differences were considered significant when *P* values were less than 0.05.

## 3. Results

### 3.1. Oxidative Stress but Not LPS-Induced RBL-2H3 Cell Degranulation Can Be Reversed by NAC and Propofol Pretreatments

**Figure 1:**
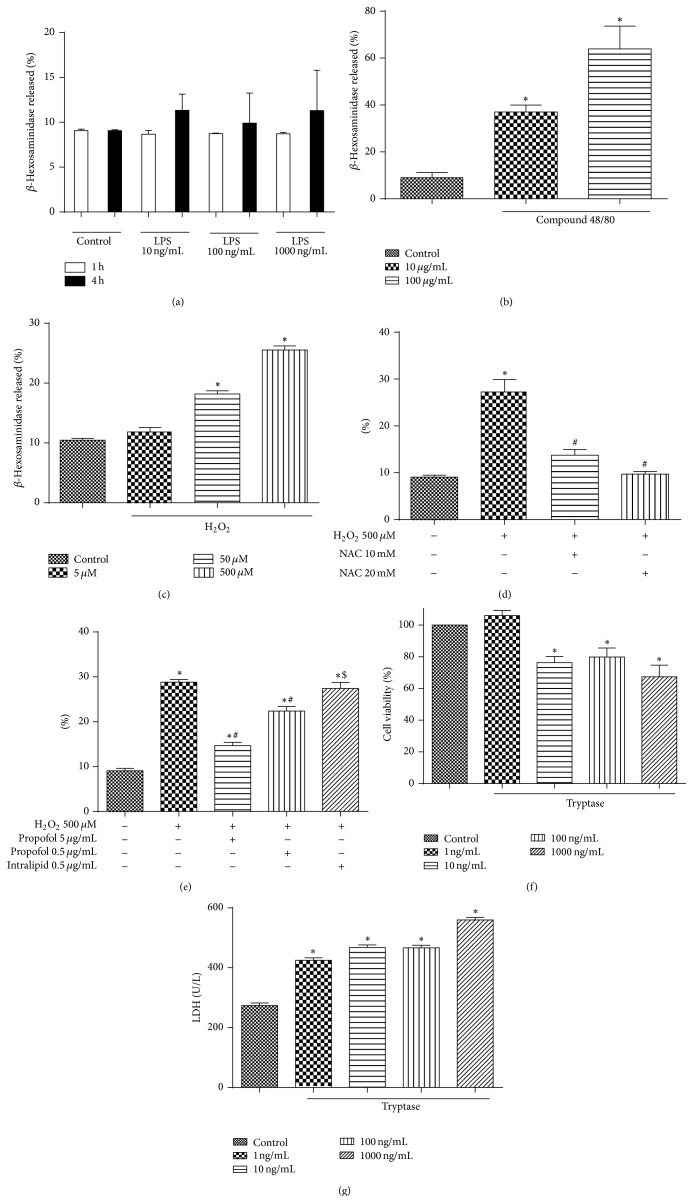
Effects of LPS, Compound 48/80, and hydrogen peroxide (H_2_O_2_) on the degranulation of RBL-2H3 cells with or without NAC or propofol treatment. (a) *β*-Hexosaminidase release after LPS treatment for 1 h or 4 h, (b) *β*-hexosaminidase release after Compound 48/80 stimulation for 1 h, and (c) *β*-hexosaminidase release after H_2_O_2_ treatment for 1 h. (d) and (e) quantified the degranulation of RBL-2H3 cells, pretreated with NAC or propofol for 12 h, induced by H_2_O_2_ stimulation for 1 h, respectively. (f) Cell viability of IEC-6 cells treated with tryptase for 12 h. Results were expressed as percentage of control group; error bars represented the standard error of the mean. (g) Lactate dehydrogenase (LDH) activity of IEC-6 cells treated with tryptase for 2 h. Results were expressed as Mean ± SEM. ^*^ *P* < 0.05 versus control group, ^#^ *P* < 0.05 versus H_2_O_2_ 500 *μ*M group, ^$^ *P* < 0.05 versus propofol 0.5 *μ*g/mL group.

Bacterial translocation and oxidative stress are the two characterizations of small IIR [37], and our previous study showed that mast cell (MC) degranulation could exacerbate the injury [14]. Therefore, in the current study, we initially sought to explore whether bacteria and oxidative stress can induce MC degranulation. As shown in Figure 1, exposure of the RBL-2H3 to different concentrations of LPS for 1 or 4 h did not cause significant changes in the release of *β*-hexosaminidase activities as compared with the control group (Figure 1(a)) whereas MC activator Compound 48/80 significantly increased the released *β*-hexosaminidase activities as compared with the control group (Figure 1(b)). This result indicates that LPS* per se* has no effects on MC degranulation at least in this experimental setting. However, treatment of RBL-2H3 cell with H_2_O_2_ dose-dependently increased the activity of the released *β*-hexosaminidase (Figure 1(c)). Pretreatments with NAC, a scavenger of oxygen radicals, abolished H_2_O_2_ mediated elevations of the released *β*-hexosaminidase activities (Figure 1(d)). Similarly, pretreatment with propofol, but not intralipid (propofol solvent), abrogated the enhancements of the released *β*-hexosaminidase activities induced by H_2_O_2_ (Figure 1(e)), indicating that oxidative stress contributes to MC degranulation and propofol inhibits MC degranulation via its antioxidant property.

### 3.2. Tryptase Contributed to Small Intestinal Epithelium Injury

Tryptase, the unique mediator released from MC degranulation [38], plays a critical role in the IIR mediated acute lung injury* in vivo* [15]. Therefore, we sought to define the direct role of tryptase in the small intestinal epithelium injury* in vitro*. A small intestinal epithelial cell line IEC-6 was employed and exposed to different concentrations of tryptase. The results showed that tryptase could directly cause IEC-6 injury in a dose-dependent manner manifested as dramatic increases in LDH activities and concomitant reductions in cell viability as compared with the control group (Figures 1(f) and 1(g)).

### 3.3. NAC, Propofol, and Intralipid Pretreatments Did Not Affect Small Intestinal Structure before IIR

**Figure 2:**
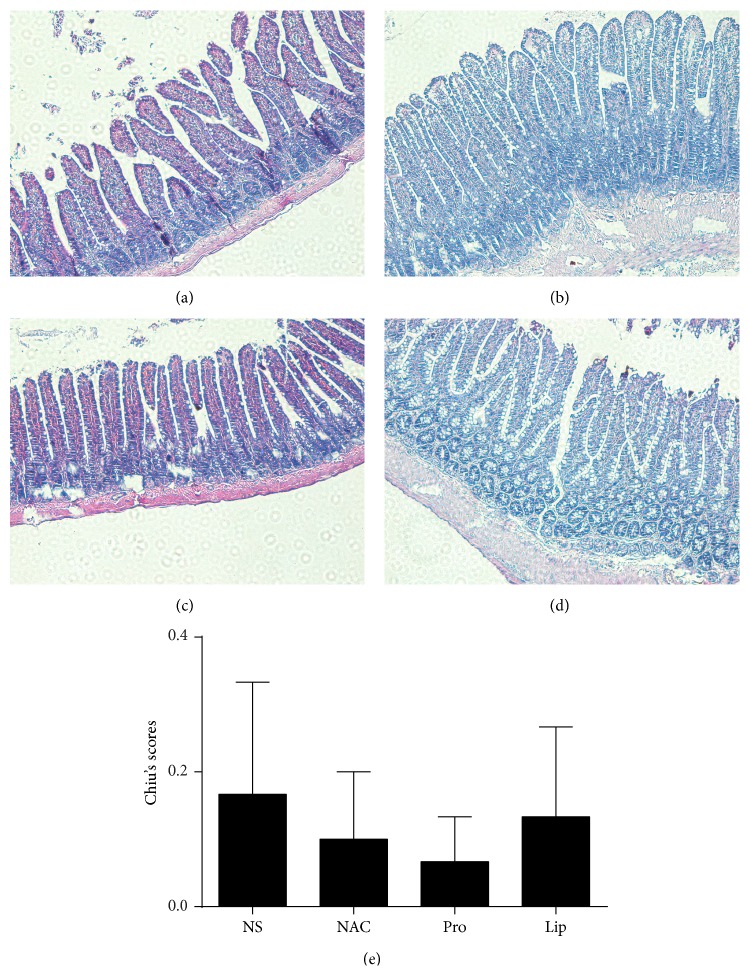
Morphological changes of intestine under light microscope in rats treated with NAC, propofol, or intralipid. ((a)–(d)) Representative images of rats treated with normal saline (NS), NAC (0.5 g/kg, i.p.), propofol (50 mg/kg, i.p.), and intralipid (50 mg/kg, i.p.) prior to ischemia reperfusion (HE staining, ×200). (e) quantified the intestine histological scores in normal saline pretreated group (NS), NAC pretreated group (NAC), propofol pretreated group (Pro), and intralipid pretreated group (Lip). Results are expressed as Mean ± SEM. *n* = 3 per group.

First, we sought to assess if pretreatment of rats with NAC, propofol, or intralipid may cause significant intestinal injury. At the dosages used, neither NAC nor propofol or intralipid caused any challenges to small intestinal structure as compared to that in rats treated with normal saline (NS). As shown in Figure 2, all the layers of intestine were normal in all the groups and there was no significant difference among groups in terms of injury score (Figure 2(d)).

### 3.4. NAC and Propofol but Not Intralipid Pretreatment Altered Intestinal Oxidant and Antioxidant Levels before IIR

**Figure 3:**
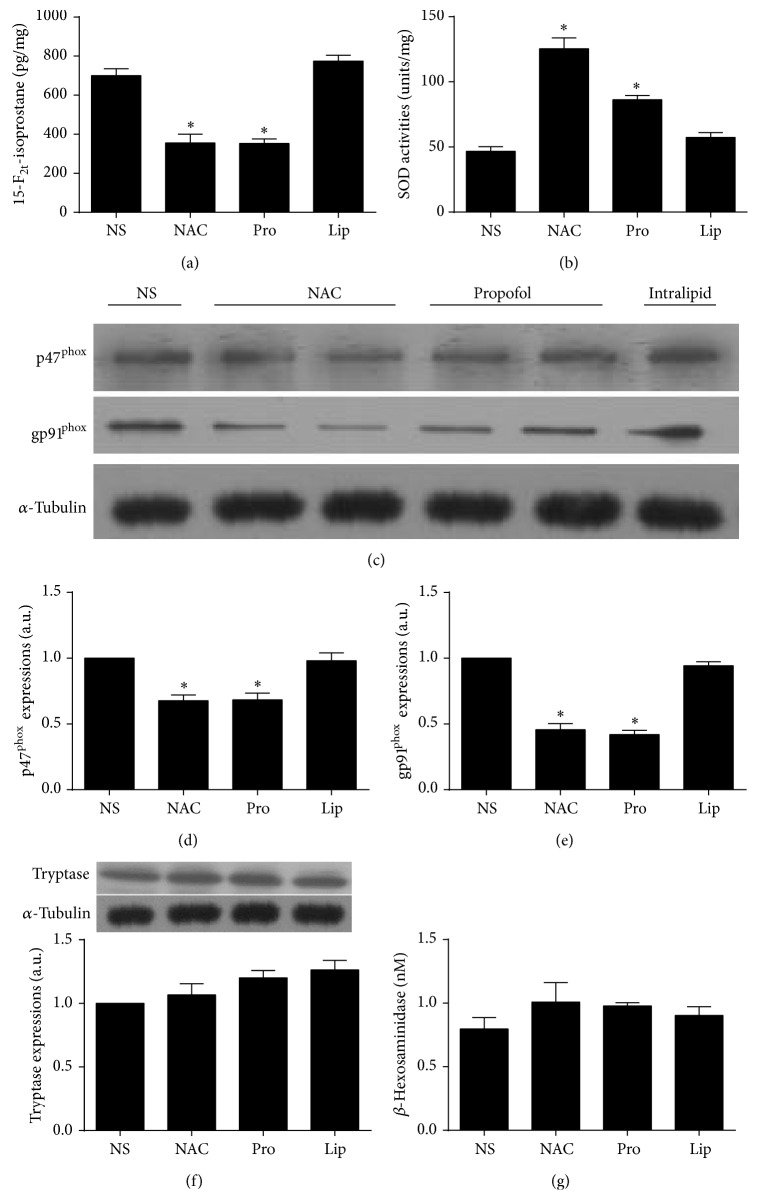
Changes of 15-F_2t_-isoprostane contents, SOD activities, p47^phox^ and gp91^phox^ protein expressions in intestine mucosa, and intestinal tryptase protein expression, and serum *β*-hexosaminidase levels in rats treated with NAC, propofol, and intralipid. (a) 15-F_2t_-isoprostane contents, (b) SOD activities, ((c)–(e)) intestinal p47^phox^ and gp91^phox^ protein expressions, (f) tryptase protein expression, and (g) *β*-hexosaminidase levels in normal saline pretreated group (NS), NAC pretreated group (NAC), propofol pretreated group (Pro), and intralipid pretreated group (Lip) (*n* = 3). Results are expressed as Mean ± SEM. ^*^ *P* < 0.05 versus NS.

NAC and propofol are well known for their antioxidative properties [10, 39]. As shown in Figure 3, NAC and propofol similarly attenuated oxidant level as demonstrated by significant decreases in 15-F_2t_-isoprostane (Figure 3(a)) contents and increases in SOD activities (Figure 3(b)) in small intestinal mucosa as compared with NS treated group. In addition, NAC and propofol pretreatment led to significant reductions in protein expressions of gp91^phox^ and p47^phox^, the important components of NADPH enzymes, in small intestinal mucosa compared with NS treated group before IIR (Figures 3(c)–3(e)). By contrast, intralipid showed comparative results to NS treated group. The data indicated that propofol, at the dose used, confers similar antioxidant effects to that of NAC.

### 3.5. NAC, Propofol, and Intralipid Pretreatments Did Not Activate Mast Cell before IIR

Trypatse and *β*-hexosaminidase are specific markers for the assessment of mast cells degranulation [40]. As shown in Figures 3(f) and 3(g), NAC, propofol, or intralipid alone had no significant effect on mast cell degranulation. There were no significant differences in tryptase protein expressions in small intestinal mucosa and *β*-hexosaminidase levels among all pretreated groups before IIR. The findings from the current study indicated that NAC and propofol pretreatments can substantially ameliorate superoxide productions without affecting mast cell and small intestine morphology under normal condition.

### 3.6. NAC and Propofol Improved Reduction of Survival Rates in Rats Challenged to IIR through Inhibiting Mast Cell Activation

**Figure 4:**
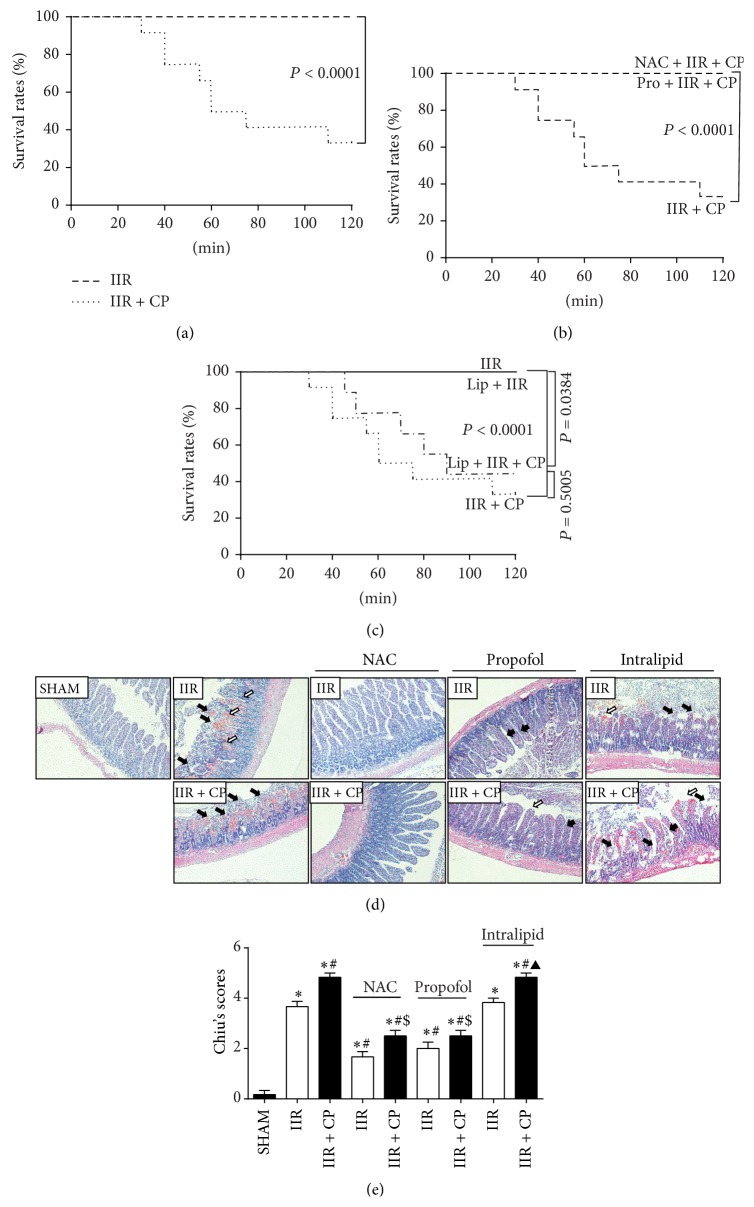
Survival rates and morphological changes of intestine and intestinal histological score under light microscope after IIR. SHAM group (Sham-operated group), IIR group (75 min intestinal ischemia and 2 h reperfusion), and IIR + CP group (IIR group + Compound 48/80 1 mg/kg) in the absence or presence of NAC (0.5 g/kg) or propofol (50 mg/kg) or intralipid (50 mg/kg). ((a)–(c)) Survival rats in rats subjected to IIR with or without NAC, propofol, or intralipid pretreatment. Results are expressed as percentage of live animals, *n* = 6 per group, whereas *n* = 12 in IIR + CP group and IIR + CP + intralipid group. (d) Representative images of HE staining (×200). Black arrow: lamina propria villus shedding. Red arrow: engorgement of capillary vessel. White arrow: top villus shedding. (e) quantified the intestine histological scores. Results are expressed as Mean ± SEM. *n* = 6 per group, whereas *n* = 4 in IIR + CP group. ^*^ *P* < 0.05 versus SHAM group, ^#^ *P* < 0.05 versus IIR group, ^$^ *P* < 0.05 versus IIR + CP group, ^&^ *P* < 0.05 versus IIR with NAC pretreated group, ^△^ *P* < 0.05 versus IIR with propofol pretreated group, and ^▲^ *P* < 0.05 versus IIR with intralipid pretreated group.

We, next, sought to investigate the effects of NAC and propofol in combating IIR and inhibiting mast cell in the process of IIR injury. As illustrated in Figure 4(a), activation of mast cell by Compound 48/80 resulted in significant decreases in 2 h survival rates after the clamp releasing as compared with sole IIR group. By contrast, NAC and propofol pretreatments showed similar promising benefits in reducing mast cell degranulation mediated injury during 2 h reperfusion period evidenced as increased postischemic 2 h survival rates to 100% (Figure 4(b)). Intralipid did not attenuate Compound 48/80 mediated exacerbation of postischemic survival rate in rats subjected to IIR (Figure 4(c)). These data suggested that oxidative stress is a major mechanism whereby mast cell degranulation exacerbated IIR injury.

### 3.7. NAC and Propofol Attenuated Small Intestinal Injury in Rats Undergoing IIR through Inhibiting Mast Cell Activation

After 2 h reperfusion, the sections of small intestine were evaluated by HE staining; IIR induced severe damage to small intestine. As depicted in Figures 4(d) and 4(e), multiple erosions and bleeding were observed in IIR group, while Compound 48/80 further aggravated IIR injury manifested as more multiple erosions and bleedings and more inflammatory cell sequestrations seen in the IIR + CP group, whereas the villus and glands were normal and no inflammatory cell infiltration was observed in mucosal epithelial layer in Sham-operated group. NAC and propofol similarly and significantly reduced the injuries in small intestine and only slight edema of mucosa villus and infiltration of few necrotic epithelial inflammatory cells were found in mucosa epithelial layer. Moreover, NAC and propofol pretreatments blocked Compound 48/80 induced exacerbation in small intestinal morphology changes after 2 h reperfusion. In contrast, pretreatment with intralipid could not attenuate IIR and Compound 48/80 induced injury. Consistent with morphological changes, Chiu's scores markedly increased in IIR group as compared with Sham-operated group while treatment with mast cell degranulator Compound 48/80 after ischemia led to further increases in Chiu's scores. NAC and propofol similarly dramatically lowered Chiu's scores and significantly limited the changes induced by Compound 48/80 (*P* < 0.05, NAC + IIR + CP or propofol + IIR + CP versus IIR + CP group). Of note, there were no differences in Chiu's scores between IIR and intralipid pretreated groups.

### 3.8. NAC and Propofol Reduced Oxidative Stress in Small Intestine in Rats Undergoing IIR through Inhibiting Mast Cell Activation

**Figure 5:**
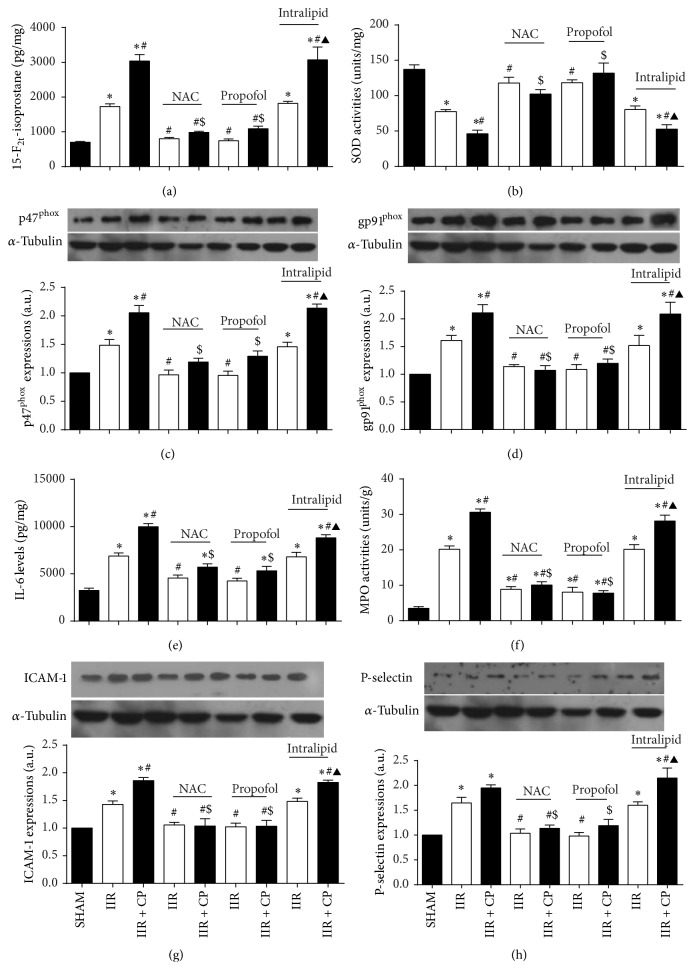
Changes of SOD activities, 15-F_2t_-isoprostane contents, p47^phox^ protein expression, gp91^phox^, P-selectin, and ICAM-1 protein expressions, IL-6 levels, and MPO activities in intestine mucosa after IIR injury. SHAM group (Sham-operated group), IIR group (75 min intestinal ischemia and 2 h reperfusion), and IIR + CP group (IIR group + Compound 48/80 1 mg/kg) in the absence or presence of NAC (0.5 g/kg), propofol (50 mg/kg), intralipid (50 mg/kg). (a) SOD activities, (b) 15-F_2t_-isoprostane contents in intestine (*n* = 6, except *n* = 4 in IIR + CP group). ((c) and (d)) p47^phox^ and gp91^phox^ protein expressions, respectively (*n* = 3), ((e) and (f)) IL-6 levels and MPO activities in intestinal mucosa (*n* = 6, except *n* = 4 in IIR + CP group). ((g) and (h)) ICAM-1 and P-selectin protein expressions, respectively (*n* = 3). Results are expressed as Mean ± SEM. ^*^ *P* < 0.05 versus SHAM group, ^#^ *P* < 0.05 versus IIR group, ^$^ *P* < 0.05 versus IIR + CP group, ^&^ *P* < 0.05 versus IIR with NAC pretreated group, ^△^ *P* < 0.05 versus IIR with propofol pretreated group, and ^▲^ *P* < 0.05 versus IIR with intralipid pretreated group.

Ischemia reperfusion injury is characterized by upregulation of ROS [10]. In line with previous results [16], we observed that 75 min ischemia followed by 2 h reperfusion led to substantial enhancements in 15-F_2t_-isoprostane contents in small intestinal mucosa and marked reductions in SOD activities as compared with Sham-operated group (Figures 5(a) and 5(b)). Meanwhile, IIR also caused great increases in gp91^phox^ and p47^phox^ protein expression when compared with Sham-operated group. Moreover, Compound 48/80 further aggravated the changes of oxidative stress induced by IIR (*P* < 0.05 IIR + CP versus IIR). Not surprisingly, propofol and NAC similarly attenuated IIR mediated oxidative stress evidenced as downregulation of 15-F_2t_-isoprostane contents and gp91^phox^ and p47^phox^ protein expression and upregulation of SOD activities in propofol and NAC treated groups as compared with group IIR. Furthermore, propofol and NAC pretreatments also limited the further increases of oxidative stress induced by Compound 48/80. As expected, intralipid did not show any antioxidative properties as the markers of oxidative stress measured were comparable between group IIR and group intralipid + IIR. Intralipid had no protective effect against Compound 48/80 induced upregulation of oxidative stress.

### 3.9. NAC and Propofol Attenuated Inflammation in Small Intestine in Rats Undergoing IIR through Inhibiting Mast Cell Activation

Interleukin 6 (IL-6) is one of the inflammatory cytokines that has been demonstrated to be implicated in the pathogenesis of IIR injury [41]. In the present study, we observed that IL-6 levels in small intestinal mucosa in group IIR were significantly increased as compared to that in the Sham-operated group (Figure 5(e)). Moreover, administration with Compound 48/80 further resulted in dramatic enhancements in IL-6 levels in group IIR + CP (*P* < 0.05 versus group IIR). Pretreatments with propofol and NAC not only similarly abrogated the increases in IL-6 levels, but also block the enhancements in IL-6 levels resulting from IIR in the presence of the MC activator Compound 48/80 (Figure 5(e)).

### 3.10. NAC and Propofol Inhibited Neutrophil Rolling in Small Intestine in Rats Undergoing IIR through Inhibiting Mast Cell Activation

IIR injury is also characterized by neutrophil sequestrating into the inflamed tissues [42]. Consistent with previous results [42], we also found that IIR led to substantial increases in MPO activities (Figure 5(f)) and ICAM-1 (Figure 5(g)) and P-selectin (Figure 5(h)) protein expressions as compared with Sham-operated group; moreover, Compound 48/80 resulted in further increases in MPO activities and P-selectin protein expression in group IIR + CP than that in group IIR. Administrations with propofol and NAC significantly inhibited neutrophil infiltration/activation evidenced as downregulation of MPO activities and P-selectin protein expression caused by IIR. Furthermore, propofol and NAC also blocked Compound 48/80 mediated exacerbation of IIR induced neutrophil infiltration.

### 3.11. NAC and Propofol Reduced Mast Cell Degranulation in Small Intestine in Rats Undergoing IIR

**Figure 6:**
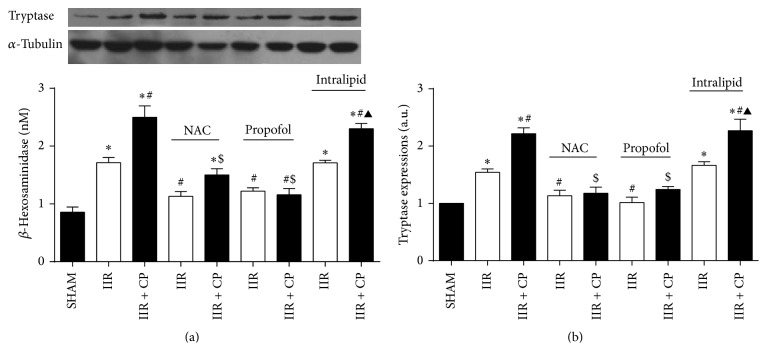
Changes of intestinal tryptase protein expression and serum *β*-hexosaminidase levels after IIR injury. SHAM group (Sham-operated group), IIR group (75 min intestinal ischemia and 2 h reperfusion), and IIR + CP group (IIR group + Compound 48/80 1 mg/kg) in the absence or presence of NAC (0.5 g/kg), propofol (50 mg/kg), intralipid (50 mg/kg). (a) Intestinal tryptase protein expression (*n* = 3). (b) *β*-hexosaminidase levels in serum (*n* = 6, except *n* = 4 in IIR + CP group). Results are expressed as Mean ± SEM. ^*^ *P* < 0.05 versus SHAM group, ^#^ *P* < 0.05 versus IIR group, ^$^ *P* < 0.05 versus IIR + CP group, ^&^ *P* < 0.05 versus IIR with NAC pretreated group, ^△^ *P* < 0.05 versus IIR with propofol pretreated group, and ^▲^ *P* < 0.05 versus IIR with intralipid pretreated group.

Mast cell releases a number of mediators that contribute to the aggravation of IIR injury, and there are many factors that can induce mast cell degranulation during IIR. Tryptase and *β*-hexosaminidase are the unique markers released from mast cell and their increased release can be recognized as mast cell degranulation. As shown in Figure 6, after 2 h reperfusion, we found that tryptase protein expression and *β*-hexosaminidase level were greatly increased in group IIR as compared with Sham-operated group, and Compound 48/80 resulted in further mast cell degranulation evidenced as dramatic increases in tryptase protein expression and *β*-hexosaminidase level observed in group IIR + CP relative to that in group IIR. Of note, propofol and NAC similarly inhibited mast cell degranulation by downregulating tryptase protein expression and *β*-hexosaminidase level triggered by IIR and Compound 48/80. Collectively, the findings from the current study indicated that oxidative stress mediated mast cell degranulation aggravated IIR injury and propofol through its antioxidative properties protects against IIR by stabilizing mast cell.

## 4. Discussion

We have shown in the current study that increased ROS during IIR exacerbated IIR injury primarily via activating mast cells evidenced as concomitant increases of postischemic 15-F_2t_-isoprostane and elevations in tryptase and *β*-hexosaminidase and increases in intestinal injury score, leading to significantly reduced postischemic survival. Further, we showed that activation of intestinal NADPH oxidase is a major source of postischemic ROS production. Antioxidant NAC and propofol inhibited postischemic intestinal NADPH oxidase activation by reducing gp91^phox^ and p47^phox^ protein overexpression and reduced ROS and the subsequent mast cell activation* in vivo* and* in vitro*, which may represent the major mechanism whereby propofol attenuates IIR injury and enhances postischemic survival.

Mast cell (MC), which contains a diverse range of mediators, resides throughout gastrointestinal tract and is also known as intestinal mucosal mast cell (IMMC). Despite the fact that MC may function as host defense to prevent bacterial invasion [43], enhanced MC activation may contribute to the development of a variety of disorders including IIR injury by releasing histamine, tryptase, TNF-*α*, and other factors. It should be noted that MC is the main source of TNF-*α* in the tract [44]. Ramos et al. reported that mast cell degranulation plays a significant role in the development of sepsis by regulating cell death, which resulted in multiorgan dysfunction [45]. Consistent with the previous studies showing that stabilizing MC from degranulation would be one of the potential strategies in combating IIR injury [14], our current study further revealed that increased oxidative stress during IIR plays an important role in mast cell activation/degranulation evidenced as enhancements in tryptase protein expression and *β*-hexosaminidase levels, which causes or exacerbates IIR injury. This notion is further supported by the fact that activating mast cell by Compound 48/80 further aggravated IIR injury and tryptase directly damaged small intestinal epithelium evidenced by the reduced cell viability and the enhanced LDH activity* in vitro*. The data from the current study further proved that mast cell activation plays a central role in exacerbating IIR injury, although the mechanism governing mast cell activation during IIR is yet to be explored.

Mast cell can be activated by several distinct mechanisms; in addition to IgE/Fc*ε*, the classical signal pathway of activation, there is a growing body of evidence to suggest that nonimmunological stimuli such as trauma and physical stress also contribute to mast cell activation [46]. Yoshimaru and colleagues demonstrated that silver mediated mast cell activation by upregulation of NADPH oxidase mediated ROS production [18]. Additionally, Collaco et al. found that superoxide production contributes to mast cell degranulation, and inhibiting ROS can stabilize mast cell* in vitro* [12]. Moreover, our previous study in a rat model of IIR revealed significant increase of mast cell activation with concomitant elevations in ROS [16]. These results provided us sound rationale to speculate that there exists a linkage between mast cell activation and superoxide production. Our current study extended findings of previous studies [16] by showing that NADPH oxidase is overexpressed during IIR which contributed to increased oxidative stress and MC activation, leading to exacerbation of IIR injury. Further, using antioxidant N-acetylcysteine (NAC), which has been shown to prevent myocardial NADPH oxidase overexpression in diabetes [47, 48] and attenuate myocardial ischemia reperfusion injury [10], we found that, in* in vivo* model of IIR, pretreatment of rats with NAC not only attenuated NADPH oxidase overexpression and reduced ROS production, but concomitantly reduced MC activation and consequently attenuated IIR injury and increased survival rate. We also found that NAC reversed the oxidative stress mediated mast cell degranulation* in vitro*. The results from the current study indicated that oxidative stress subsequent to NADPH oxidase overexpression during IIR plays a critical role in mast cell activation, which in turn led to a deleterious injury. Of note, in the current study, mast cell activation by CP also induced oxidative stress manifested as increased 15-F_2t_-isoprostane and reduced SOD, associated with increased p47^phox^ and gp91^phox^ protein expression in IIR + CP group compared to IIR group (Figures 5(a)–5(d)). This is consistent with study from Collaco et al. showing that sodium sulfite activated mast cell degranulation and subsequently increased intercellular oxidative stress in RBL-2H3 cells [12]. These indicate that mast cell activation* per se* induces oxidative stress.

Excessive production of ROS by NADPH oxidase is generally considered to be involved in the pathogenesis of inflamed tissues [49], including ischemic tissues. There are a number of NADPH homologs presented in many diverse organs, such as Nox1, Nox2 (also named as gp91^phox^), and Nox3-4 [50]. It is important to appreciate that Nox2 is predominantly expressed within epithelial cells. In addition to ROS scavenger, NAC also inhibits NADPH oxidase activities; our previous results showed that pretreatment of diabetic rats with NAC attenuated cardiac ischemia reperfusion injury by downregulating NADPH subunits p67^phox^ and p22^phox^ expression and reducing oxidative stress [48]. Inoue et al. have demonstrated that blockade of ROS generation by NADPH oxidase inhibition can limit mast cell degranulation [51]. Guan et al. demonstrated that intracellular NADPH concentration in villus tip cells in intestine was significantly rapidly increased even after short-term ischemia [52]. In the current study, we also found that elevations of gp91^phox^ and p47^phox^ protein expression are the prominent features of IIR. Furthermore, precondition of IIR-rats with antioxidant NAC or propofol not only significantly reduced gp91^phox^ and p47^phox^ protein expression, but also played a significant role in stabilizing mast cell.

Propofol is a short-acting, intravenously administered hypnotic agent. In addition to its sedation/hypnotic properties, propofol displays protective effects in many organs subjected to ischemia reperfusion injury by inhibiting oxidative cellular damage [53, 54]. Hama-Tomioka et al. recently reported that propofol can block superoxide production originated from NADPH oxidase* in vitro* [55]. Although, as described previously, a single intraperitoneal injection of 50 or 100 mg/kg propofol could significantly attenuate IIR injury in acute rat models [31], propofol is the most commonly used agent overall during short-term and intermediate-length sedation in ICU [56]; thus, in the current study, we explored propofol pretreatment for three successional days and incorporated the investigation of postischemic mortality in addition to observing its effect on IIR injury. To our knowledge, our study is the first to show that propofol pretreatment given at a sedative dosage significantly inhibited the overexpression of NADPH oxidase and reduced oxidative mediated MC action during IIR or* in vitro* and reduced IIR injury and subsequently enhanced postischemic mortality in rats.

IIR injury is also characterized by uncontrollable inflammation and neutrophils sequestration in inflamed tissues. Migration of neutrophils to injurious site is mediated by selectins and intercellular adhesion molecules (ICAMs) by the activated endothelium [57]. Compton et al. reviewed that tryptase released from mast cell degranulation can attract leukocytes infiltrate and migrate to ischemic tissues [58]. And treatment of rats with mast cell stabilizer cromolyn sodium has been shown to greatly reduce the expressions of ICAM-1 in the lungs in pancreatitis-associated lung injury and decreased IL-6 release [59]. These findings point to the importance of mast cell degranulation through increased release of proinflammatory mediator in inducing neutrophil migration to inflamed tissues. In the present study, we found that, during IIR, mast cell degranulation resulted in more neutrophils infiltration into small intestine evidenced as significant increases in MPO activities and ICAM-1 and P-selectin protein expressions and that propofol and NAC similarly blocked the alterations induced by IIR and prevented MC stimulator Compound 48/80 mediated exacerbation of IIR injury. Our finding that propofol can inhibit intestinal MC activation during IIR and reduce tissue neutrophils infiltration as well as the subsequent systemic inflammation may have potential clinical importance given that IIR occurs often in patient who underwent major surgeries such as cardiac surgery with cardiopulmonary bypass [60] and is a challenging and life-threatening clinical problem. The delay in diagnosis and treatment of IIR injury contributes to the continued high in-hospital mortality rate [61]. Preventive administration of propofol may be a promising approach in attenuating postoperative intestinal ischemia and mortality.

In a summary, we have shown that mast cell activation, through increased release of mediators, contributed to deleterious injury induced by IIR and that oxidative stress plays a central role in mast cell activation. Propofol, similar to the antioxidant NAC, inhibited ROS mediated mast cell activation and attenuated IIR injury and enhanced postischemic mortality. Propofol mediated reduction of oxidative stress and MC activation during IIR is achieved at least in part through attenuating IIR induced NADPH oxidase overexpression, while the underlying mechanism merits further study. Mast cell activation contributes to and exacerbates small intestinal ischemia reperfusion injury.
