# Corrigendum to “Propofol Attenuates Small Intestinal Ischemia Reperfusion Injury through Inhibiting NADPH Oxidase Mediated Mast Cell Activation”

**DOI:** 10.1155/2017/8932871

**Published:** 2017-08-14

**Authors:** Xiaoliang Gan, Dandan Xing, Guangjie Su, Shun Li, Chenfang Luo, Michael G. Irwin, Zhengyuan Xia, Haobo Li, Ziqing Hei

**Affiliations:** ^1^Department of Anesthesiology, The Third Affiliated Hospital, Sun Yat-sen University, Guangzhou 510630, China; ^2^Zhongshan Ophthalmic Center, Department of Anesthesiology, Sun Yat-sen University, Guangzhou 510060, China; ^3^Department of Anesthesiology, University of Hong Kong, Hong Kong

In the article titled “Propofol Attenuates Small Intestinal Ischemia Reperfusion Injury through Inhibiting NADPH Oxidase Mediated Mast Cell Activation” [1], the *α*-tubulin samples in Figures 5(g) and 5(h) should have included nine samples; however, due to an error during the production process, only seven samples appeared. Also, the legends of figures 5A and 5B were reversed. The corrected figures and legend are as follows.

## Figures and Tables

**Figure 5:**
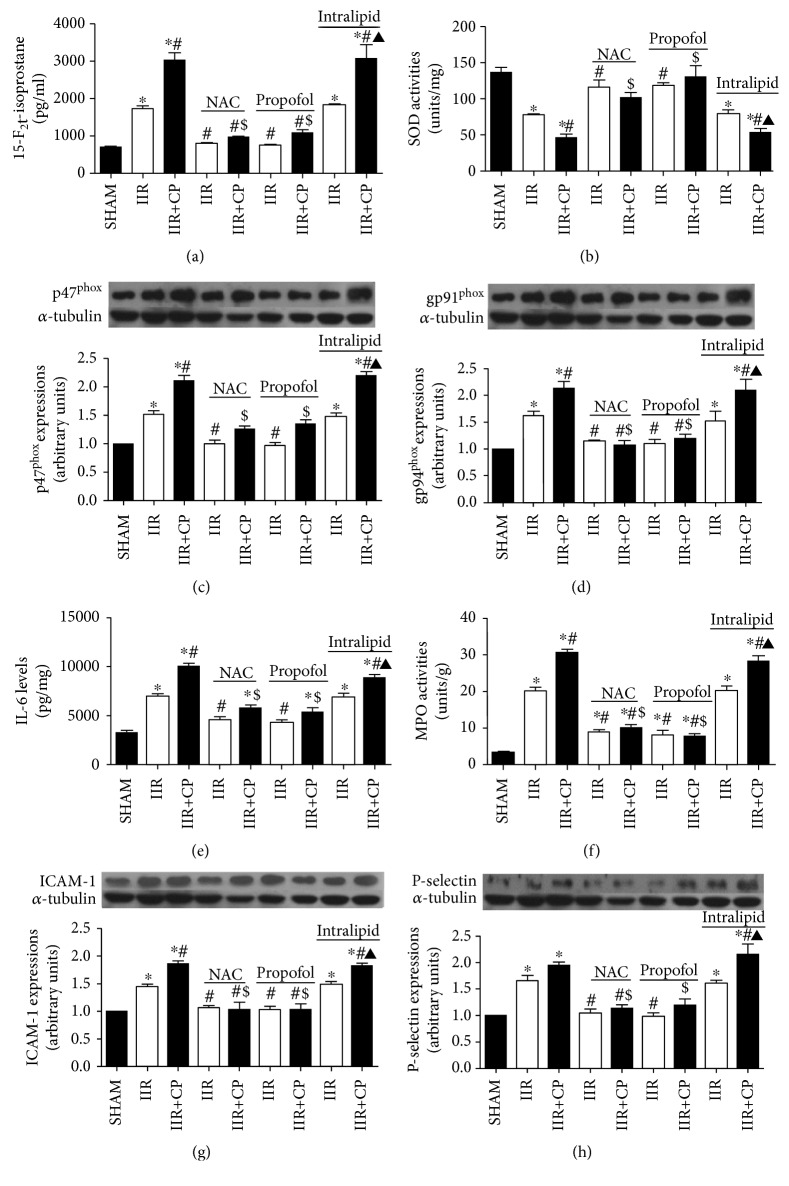
Changes of SOD activities, 15-F_2t_-isoprostane contents, p47^phox^ protein expression, gp91^phox^, P-selectin, and ICAM-1 protein expressions, IL-6 levels, and MPO activities in intestine mucosa after IIR injury. SHAM group (Sham-operated group), IIR group (75 min intestinal ischemia and 2 h reperfusion), and IIR + CP group (IIR group + compound 48/80 1 mg/kg) in the absence or presence of NAC (0.5 g/kg), propofol (50 mg/kg), intralipid (50 mg/kg). (a) 15-F_2t_-isoprostane contents in intestine (*n* = 6, except *n* = 4 in IIR + CP group). (b) SOD activities (c) and (d) p47^phox^ and gp91^phox^ protein expressions, respectively (*n* = 3), (e) and (f) IL-6 levels and MPO activities in intestinal mucosa (*n* = 6, except *n* = 4 in IIR + CP group). (g) and (h) ICAM-1 and P-selectin protein expressions, respectively (*n* = 3). Results are expressed as mean ± SEM. ^∗^*P* < 0.05 versus SHAM group, ^#^*P* < 0.05 versus IIR group, ^$^*P* < 0.05 versus IIR + CP group, ^&^*P* < 0.05 versus IIR with NAC pretreated group, ^△^*P* < 0.05 versus IIR with propofol pretreated group, and ^▲^*P* < 0.05 versus IIR with intralipid pretreated group.

## References

[1] Gan X., Xing D., Su G. (2015). Propofol attenuates small intestinal ischemia reperfusion injury through inhibiting NADPH oxidase mediated mast cell activation. *Oxidative Medicine and Cellular Longevity*.

